# In pursuit of increasing the application of tele-audiology in South Africa: COVID-19 puts on the alert for patient site facilitator training

**DOI:** 10.4102/sajcd.v69i2.900

**Published:** 2022-07-20

**Authors:** Katijah Khoza-Shangase

**Affiliations:** 1 Department of Audiology, Faculty of Humanities, University of the Witwatersrand, Johannesburg, South Africa

**Keywords:** audiology, COVID-19, human resources, patient site facilitators, South Africa, tele-audiology, training

## Abstract

**Background:**

The coronavirus disease 2019 (COVID-19) presented and highlighted new and unanticipated challenges to the provision of clinical services, raising an urgency for the application of different models of service delivery, including tele-audiology. In many tele-audiology encounters, a site facilitator is needed at the patient site to help with the hands-on aspects of procedures, and the implications of this requirement are significant for the resource-constrained African context.

**Objectives:**

The aim of this scoping review was to investigate published evidence on training provided to patient site facilitators (PSFs) for tele-audiology application to guide the South African audiology community in tele-audiology application initiatives.

**Method:**

Electronic bibliographic databases including Science Direct, PubMed, Scopus MEDLINE and ProQuest were searched to identify peer-reviewed publications, published in English, between 2017 and 2021 related to training of PSFs. The guidelines of the Preferred Reporting Items for Systematic Reviews and Meta-Analysis (PRISMA) were followed during the screening process as well as for illustrating the process.

**Results:**

Findings are discussed under four key themes: (1) type of tele-audiology and the implications thereof, (2) length of training and its implications, (3) diversity in the range of PSFs used and its implications for the training, and (4) heterogeneity in the training.

**Conclusion:**

The findings highlight important considerations for tele-audiology application within the African context, specifically decision-making around who can serve in the role of PSFs, as well as content and nature of training required, with implications for policy and regulations as well as human resource strategy. These findings are important for the COVID-19 pandemic era and beyond.

## Introduction

The need for, and therefore access to, ear and hearing services is undisputed globally. The World Health Organisation (WHO, 2018) provides estimates of disabling hearing loss to be over 6.1% of the global population (466 million individuals), with statistics indicating that these numbers will rise over the years, with estimates of up to 630 million by 2030 and above 900 million in 2050. Closer to home, the prevalence of hearing loss is documented to be higher in sub-Saharan Africa than in other regions of the world (Mulwafu, Kuper, & Ensink, 2016; Wonkam Tingang et al., 2020), with South Africa reported to have 4 million deaf and hard of hearing people (Western Cape Government, 2020). Evidence suggests that these numbers will rise over the coming decades with hearing loss linked to the burden of diseases most prevalent in this context such as tuberculosis (TB), human immunodeficiency virus (HIV) (Christopher, Edward & Sabrina, & Agnes, 2013; Khoza-Shangase, 2010, 2017, 2020; Tshifularo, Govender, & Monama, 2013) and presbycusis influencing the prevalence. Farmer et al. (2010) also predict that 70% of cancers will occur in low-and-middle-income countries (LMICs) by 2030, including cancers of the ear, nose and throat, where ototoxicity linked to chemotherapeutic agents become a concern. All these factors, including the possible and documented impact of the coronavirus disease 2019 (COVID-19), long COVID and its treatments on cochleovestibular function in adults (Fancello et al., 2021; Jacob, Flannery, & Mostert, 2020; Koumpa, Forde, & Manjaly, 2020; Munro, Uus, Almufarrij, Chaudhuri, & Yioe, 2020), highlight a strong need for careful planning around innovative models of ear and hearing healthcare service provision in sub-Saharan Africa. This is also applicable to other similar LMIC contexts, where capacity versus demand challenges as far as availability of audiologists is concerned and is an additional stress to ear and hearing care (Khoza-Shangase, 2021; Pillay, Tiwari, Kathard, & Chikte, 2020).

The Speech-Language and Hearing Professions Board of the Health Professions Council of South Africa (HPCSA) strongly advocates for best-practice guided assessment and intervention services, within service delivery models that are contextually, linguistically and culturally congruent with the South African context and population (Khoza-Shangase, 2020a). This best practice includes the use of telehealth and mobile practice in rendering clinical services. Literature on tele-audiology in South Africa has focused primarily on reaching many people who live in underserved areas (Dawood, Mahomed Asmail, Louw, & Swanepoel, 2020; Sandström, Swanepoel, Laurent, Umefjord, & Lundberg, 2020; Van Wyk, Mahomed-Asmail, & Swanepoel, 2019). Given that South African audiological practice is still based on Euro-Western epistemology and ideology (Khoza-Shangase & Mophosho, 2018, 2021), there is a need for careful considerations and deliberations around the delivery of audiological services through telehealth systems, including the use of and the role of patient site facilitators (PSFs), who are linguistically and culturally diverse and have been appropriately trained for the context, with this approach potentially enhancing contextual relevance of the services provided.

The well-documented challenges relating to the availability of audiologists for the size of the population requiring ear and hearing care (capacity versus demand), as well as the advent of COVID-19 and its direct patient interaction challenges, over and above the vestibulocochlear signs and symptoms linked to it (Khoza-Shangase, in press), call for a serious re-imagining of the human resource strategy as well as the service delivery model for ear and hearing care within the South African context (Khoza-Shangase & Masondo, 2021; Muñoz, Nagaraj, & Nichols, 2020; Saunders & Roughley, 2021; Sebothoma, Khoza-Shangase, Masege, & Mol, 2021). Key to this re-imagining exercise is a paradigm shift that embraces the application of innovative service delivery models with the inclusion of paraprofessionals (Khoza-Shangase, 2021). The practical use of existing and emerging technologies for provision of clinical services as part of tele-practice has been documented to (1) increase and expand access to specialised expertise that is otherwise not readily available, (2) enrich clinicians’ efficiency and output and (3) increase access to quality services whilst maintaining costs (Khoza-Shangase & Sebothoma, 2022). Furthermore, telepractice in the form of tele-audiology within the South African context opens job opportunities where paraprofessionals are utilised in task-shifting roles where they serve as PSFs. Within audiology, tele-audiology can create such job opportunities where people with no background in audiology such as family members, community members and others can have an opportunity to receive training on new skills as PSFs and be employed in ear and hearing care programmes that are instituted remotely. However, successful, ethical and clinical service and patient-safe tele-audiology service provision requires effective and efficient training and management of PSFs.

Although telepractice has been around for over two decades (Kim et al., 2020), its application has gained much popularity during the COVID-19 pandemic (Sebothoma et al., 2021). Due to the virus’s highly infectious nature, non-pharmaceutical measures to prevent its spread had to be implemented, and these significantly affected person-to-person contact for clinical care provision (Khoza-Shangase, Moroe, & Neille, 2021). National lockdowns with accompanying travel restrictions, regulations around social distancing, isolation and quarantining, hand washing and sanitisation, as well as community containment remain the only measures available (because efficacy of vaccines and vaccine access and hesitancy remain a challenge), and these have a measurable effect on clinical service provision (Department of Co-operative Governance and Traditional Affairs, 2020; Khoza-Shangase et al., 2021; Perez et al., 2020). Kim et al. (2020) argue that these measures influenced the increased need for telepractice services, with tele-audiology falling within these services (a). Tele-audiology allows audiologists to have access to patients in remote areas, over considerable distances, without compromising the validity and reliability of the service being provided, whilst affording patients easier access to an even wider range of ear and hearing healthcare services (Kim, Jeon, Kim, & Shin, 2021; Ratanjee-Vanmali, Swanepoel, & Laplante-Levesque, 2019).

In low-and-middle-income countries (LMICs) such as South Africa, provision of ear and hearing care services is confronted by numerous challenges, with capacity versus demand challenges being one key difficulty. Tele-audiology is therefore an opportunity requiring much more thoughtful and deliberative consideration (Khoza-Shangase et al., 2021), with COVID-19 having made this need starkly obvious (Swanepoel & Hall, 2020). Its application within these contexts remains at its infancy, with limited evidence in the use of PSFs as part of the service delivery model. Although tele-audiology offers numerous well-documented benefits, there are also a few challenges that might be encountered in its application, particularly in the African context. These challenges became pronounced during the COVID-19 pandemic as large-scale application had to be applied. Besides lack of sufficient knowledge and skills in tele-practice by audiologists (Ravi, Gunjawate, Yerraguntla, & Driscoll, 2018; Sebothoma & Khoza-Shangase, 2021), access to the required infrastructure and technology by both patients and clinicians as well as software information and technical limitations (Molini-Avejonas, Rondon-Melo, De La Higuera Amato, & Samelli, 2015; Ravi et al., 2018; Sebothoma et al., 2021), good internet connectivity (Wolfgang, 2019), reimbursement and licensure barriers (Ravi et al., 2018) and lack of reliable electricity supply within the South African context are some of the challenges encountered. For the focus of the current paper, access to PSFs who are skilled and able to facilitate the provision of a tele-audiology service can be a significant barrier, which became highlighted by the COVID-19 pandemic within the South African audiology clinical context, with this role mostly being filled by audiologists and/or audiology students (Sebothoma et al., 2021).

Most tele-audiology services require the presence of a PSF at the patient site to assist with technical support and the hands-on aspects of the tele-audiology session, such as patient positioning and orientation to the equipment for the duration of the appointment, as well as assisting with instructions and general communication with the patient where necessary (Coco, Davidson, & Marrone, 2020; Krumm, 2016; Wolfgang, 2019). Coco et al. (2020) also refer to PSFs as patient-site presenters, telepresenters, assistants and e-helpers, and they maintain that even in the presence of advanced technology, PSFs are still very helpful in ensuring provision of the best services and in increasing the efficiency of tele-audiology. Furthermore, Lancaster, Krumm, Ribera and Klich (2008) argue that properly trained PSFs are probably the most critical component of telehealth applications, and yet no internationally documented or available standard of training exists.

Although it is clear that PSFs are key characters in successful application of tele-audiology within the South African context, no standard nor regulated training programme exists for these cadres, nor do minimum standards exist for who can get trained and what constitutes the training, unless this becomes part of the job description of the newly formalised HPCSA audiology assistants’ minimum standards and regulations (HPCSA, n.d.). These, however, are yet to be implemented (Moroe, personal communication, HPCSA Professional Board). South African studies on the application of tele-audiology reveal diverse practices, including varied individualised training, use of either nurses or audiology students and use of PSFs for varied functions, depending on what the study was investigating (Mahomed-Asmail, Swanepoel, & Eikelboom, 2016; Sebothoma et al., 2021; Yousuf Hussein, Swanepoel, Mahomed, & Biagio De Jager, 2018). This is in line with the findings by Coco et al. (2020) where PSFs were found to come from various backgrounds and perform a variety of duties. With the range of potential and recorded duties of PSFs as well as the diversity of individuals who can perform these duties, the training of these individuals becomes paramount for successful, safe and ethical tele-audiology practice, hence the purpose of this scoping review aimed at documenting published evidence on the training provided to PSFs for tele-audiology application, in order to guide the South African audiology community in tele-audiology application initiatives.

## Methodology

### Aim

The aim of this scoping review was to document published evidence on the training provided to PSFs for tele-audiology application.

### Review approach

Based on a definition of a scoping review by Munn et al. (2018), this review, first of its kind looking at this specific question, used the Arksey and O’Malley’s (2005) framework for conducting a scoping review and adhered to the following stages of conducting a review: (1) specifying the research question, (2) identifying the relevant literature, (3) selecting studies to be included in the review, (4) mapping out the data, as well as (5) summarising, synthesising and reporting the results. The guidelines of the Preferred Reporting Items for Systematic Reviews and Meta-Analysis (PRISMA) were followed during the screening process as well as for illustrating the process.

#### Stage 1: Identification of the research question

The population (PSFs), concept (training of PSFs) and context (in tele-audiology) Population (or participants), Concept, Context (PCC) framework by the Joanna Briggs Institute was used to formulate the research question. For the current review, the following question was formulated: ‘What training is provided to PSFs for tele-audiology application?’.

#### Stage 2: Data sources and search strategy

The literature search was conducted in December 2021 on databases including ProQuest, PubMed, Scopus, Science Direct and MEDLINE. To identify papers relevant for this review, the following terms and combination of keywords were used: tele-audiology training, as well as training of PSFs AND tele-audiology, telehealth, tele-audiology, PSFs, tele-audiology AND facilitators, PSFs AND training. Other websites such as South African Speech Language and Hearing Association (SASLHA) and the HPCSA website were searched to identify any grey literature, as well as the reference lists of the screened papers. Ghalibaf (2017) says that using a combination of keywords increases the likelihood that the search yields specific and relevant papers and eliminates the irrelevant studies.

#### Inclusion criteria

To yield contemporary evidence, the search was restricted to recent studies published in English not more than 5 years ago (between the year 2017 and 2021). To form part of the review, the studies had to be peer-reviewed, based on tele-audiology, include the use of PSFs and describe the training of the PSFs.

#### Exclusion criteria

Studies were not eligible for inclusion if they were published more than 5 years ago, as the study was deliberately investigating recent contemporary evidence, and if they were not published in English. It was very important to include studies in the past 5 years to accommodate the possible impact of both recent technological advances in audiology as well as COVID-19. Studies were excluded if they did not address the research question and if they did not include the combination of the search keywords. Studies that spoke about PSFs but were not in a tele-audiology setting were also excluded.

#### Stage 3: Study selection

**FIGURE 1:**
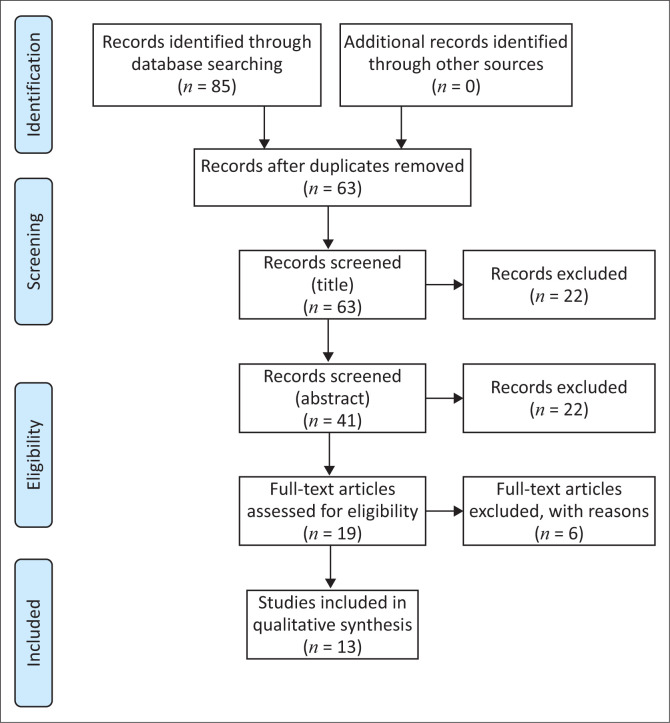
PRISMA 2020 flow diagram for the current scoping review which included searches of databases and other sources.

In total, the database search from all databases identified 85 articles. All identified database citations were exported to Endnote, a web-based bibliography manager. Through Endnote, duplicate studies were identified and removed. After the duplicates were removed, 63 studies remained, and of these, 22 were excluded at title screening. Following this step, 41 abstracts were screened guided by the search question. Of these, 22 studies were excluded due to not being related to tele-audiology. Nineteen studies were then assessed for eligibility; of these, six were excluded because they did not include PSF training. Consequently, a full-text screening resulted in 13 studies meeting the inclusion criteria and being included in this scoping review. Figure 1 shows a PRISMA flowchart for the literature search, retrieval and inclusion process of this scoping review.

#### Stage 4: Data extraction and charting

After the reference search, a total of 13 studies were included in this review, and these are depicted in Table 1. The table included the following details about the included articles, with detailed analysis and discussion of the contents of the table presented next:

- Researcher/s and the year of publication
- Title of the study
- Country of publication
- Type of tele-audiology
- Patient site facilitator
- PSF duties
- PSF trainer
- Duration of facilitator training
- Content of the training

**TABLE 1:** Summary of studies included in the scoping review documenting the training of patient site facilitators.

	Researcher/s & Year of publication	Study title	Country	Type of tele-audiology	Patient site facilitator	Patient site facilitator duties	Patient site facilitator trainer	Duration of training	Content of the training
**1**	Lundberg, De Jager, Swanepoel and Laurent (2017)	Diagnostic accuracy of a general practitioner with video-otoscopy collected by a health care facilitator compared to traditional otoscopy.	South Africa	Synchronous	Healthcare facilitator	Capture video otoscopic images	An Otologist	2 days	How to conduct video otoscopy.
**2**	Monica et al. (2017)	School entry level tele-hearing screening in a town in South India–Lessons learnt.	India	Synchronous	Teacher	Perform video otoscopy, hearing screenings and Ling Six sound tests.	Audiologist	2 days	Technical knowledge such as ensuring proper connectivity of the computer hardware, the internet and placement of transducers on the child’s head, insertion of OAE probe, infection control
**3**	Ramkumar, Vanaja, Hall, Selvakumar and Nagarajan (2018)	Validation of DPOAE screening conducted by village health workers in a rural community with real-time click evoked tele-auditory brainstem response.	India	Synchronous	Village health workers	Appropriate positioning of patient, ensuring the child is asleep during ABR testing and ABR electrodes placement	No details	5 days	How to conduct and read DPOAE screening.
**4**	Govender and Mars (2018)	Assessing the efficacy of asynchronous telehealth-based hearing screening and diagnostic services using automated audiometry in a rural South African school.	South Africa	Asynchronous	Community member	Conduct video otoscopy and audiometric screening via an automated protocol, set up equipment and instruct patients on testing procedures	Academics/Audiologists	2 days	An overview of the hearing screening protocol with a practical component, orientation of equipment, patient management, troubleshooting.
**5**	Hughes, Goehring, Sevier and Choi (2018)	Measuring sound-processor thresholds for pediatric cochlear implant recipients using visual reinforcement audiometry via telepractice.	United States of America	Synchronous	Audiologist or speech pathologist	Connect cochlear implant processor to the programming cable, focusing the child’s attention through using toys.	Audiologist	No details	Observing recorded clinical sessions and receiving instructions on how to perform the procedures.
**6**	Venail et al. (2018)	Evaluation of otoscopy simulation as a training tool for real-time remote otoscopy.	France	Synchronous	Third year undergraduate students with no previous experience of eardrum examinations	Conduct video otoscopy and identify landmarks on the eardrum.	No details	2 h	Anatomy of the external and middle ear, training with otoscopy simulator focused on hand positioning and proper software use.
**7**	Erkkola-Anttinen et al. (2019)	Smartphone otoscopy performed by parents.	Finland	Asynchronous	Parents	Conduct smartphone otoscopy and send the videos to the physician, and if necessary, take the child to the physician.	Physician	1 h	How to conduct smartphone otoscopy.
**8**	Hatton, Rowlandson, Beers and Small (2019)	Telehealth-enabled auditory brainstem response testing for infants living in rural communities: The British Columbia Early Hearing Program experience.	Greenland	Synchronous	Audiometric technician	Greeting the patients and preparing them for ABR testing by placing electrodes and transducers.	Audiologist	3–4 days	How to conduct an ABR.
**9**	Eksteen et al. (2019)	Hearing and vision screening for preschool children using mobile technology, South Africa.	South Africa	Synchronous	Community health worker	Perform hearing screenings using a mobile device.	Audiologist	5 days	How to conduct hearing screening, how to use equipment and how to evaluate the responses.
**10**	Ravi et al. (2020)	Tele-audiological surveillance of middle ear status among individuals with cleft lip and/or palate in rural South India.	India	Asynchronous and synchronous	Community-based rehabilitation workers	Perform video otoscopy and set up for videoconferencing sessions, placing transducers on patients.	Audiologist	No details	Operating the laptop and ensuring internet connectivity using mobile hotspot, setting up the microphone and web camera for videoconferencing, how to connect audiological equipment to the laptop, placing transducers on patients.
**11**	Coco et al. (2020)	The role of patient-site facilitators in teleaudiology: A scoping review	Not applicable	-	Fourteen categories of individuals who serve as facilitators, e.g. nurses, students, community healthcare workers, etc.	Audiology related duties such as: scrubbing patients for ABR, performing the Dix Hallpike manoeuvre, Ling six test, screenings, etc. General duties such as: sending data from the patient site, sanitizing the test area, obtaining patient consent.	-	-	Majority of the studies included did not specify training.
**12**	Tao et al. (2021)	Teleaudiology hearing aid fitting follow-up consultations for adults: Single blinded crossover randomised control trial and cohort studies.	Australia	Synchronous	Unspecified facilitators	Hands-on tasks such as demonstrating how to insert and remove the hearing aids, inspecting the ear, repeating the audiologist’s message if necessary and other tasks required by the clinician.	No details	2 days	Using a laptop and software to connect remotely, handling and manipulation of hearing aids, earpieces, and hearing aid accessories, how to grind and drill ear moulds, how to inspect the ear and ear canal, orientation and proper insertion and positioning of hearing aids, use of hearing aid accessories (e.g. otoscope, domes, cerustops, magnets, rotary tool for drilling and grinding, cleaning kit, receivers, slim tubes).
**13**	Coco, Piper and Marrone (2021)	Feasibility of community health workers as teleaudiology patient-site facilitators: A multilevel training study.	United States of America	Synchronous	Community health workers	Teleaudiology services	Researcher/audiologist experienced in hearing loss and teleaudiology training.	Introductory level: 1 h Intermediate level:1.5 h Facilitator level:12 h over 2 days	Three training levels: (1) introductory level, basic information about causes and effects of hearing loss and basic tele-audiology and its benefits; (2) intermediate level, technology, roles of the tele-audiology service delivery team, tele-audiology technology and patient safety and confidentiality; (3) facilitator level, knowledge and hands-on skills required to serve as patient-site facilitators, a 4-h in-person observation at a university-based adult audiology clinic, which included hearing tests, hearing aid consultations, initial hearing aid fittings and follow-up care for troubleshooting appointments.

#### Stage 5: Collating, summarising, and reporting results

As suggested by Levac, Colquhoun and O’Brien (2010), this stage was separated into three phases, namely, the analysis, reporting overall results and the implications phase. Thematic analysis was adopted when analysing the evidence and findings reported in accordance with the stated aim of the study. Lastly, the implications of current findings are raised for training of PSFs for the South African context’s tele-audiology application initiatives.

### Ethical considerations

In this scoping review, ethical standards were adhered to and there were no risks as there were no human participants involved. Common forms of search biases, such as database bias, country bias, availability bias, familiarity bias and multiple publication bias, as advocated by Suri (2020), were considered.

## Results and discussion

Analysis of results raises four key themes, and these will be presented within a narrative discussion below: (1) type of tele-audiology and the implications thereof, (2) length of training and its implications, (3) diversity in the range of PSFs used and its implications for the training and 4) heterogeneity in the training.

### Type of tele-audiology and the implications thereof

As depicted in Table 1, firstly, the 13 studies included in the current review revealed a significant preference for synchronous or live face-to-face tele-audiology, with three papers reporting on the application of the asynchronous model (Erkkola-Anttinen et al., 2019; Govender & Mars, 2018; Ravi et al., 2020). This is a challenge as this model truly and mainly addresses the geographic challenge as a driving force for tele-audiology application and not necessarily the workforce challenge, which South Africa also confronts. The fact that the audiologist needs to be physically there in real time via video connection between them and the patient, with the PSF being required for ‘hands-on’ tasks, limits the possibilities of access because the audiologist needs to be present and running the consultation (Bennett, Swanepoel, Manchaiah, & Eikelboom, 2020). In LMICs, there must be serious considerations of asynchronous tele-audiology applications as well. The fact that most of the evidence on the training of PSFs is based on the synchronous model of application should therefore be held in cognisance, and planning should consider the asynchronous model for the African context. With the asynchronous (store-and-forward) tele-audiology model, the audiologist does not need to be present for the consultation, but the patient and/or PSF does. In the South African context, the current author recommends that the audiology technician ‘runs’ the programme to obtain results that then get stored and forwarded to the audiologist for interpretation, synthesis, and clinical decision making (Bennett et al., 2020). Consequently, training of PSFs (or in the South African context, possibly audiology technicians) would need to be more intensive for the asynchronous tele-audiology model and the list of duties more comprehensive, and in both models, duties would need to be very well defined.

In the South African context, audiology technicians are mid-level workers for whom the South African National Department of Health has been liaising with the HPCSA about developing minimum standards and regulations, in order to increase access to ear and hearing care services in South Africa. Although these cadres are not in existence yet, an HPCSA (n.d.) *Guideline for planning STA (Speech Therapy and Audiology) services at all levels of health care* in South Africa clearly specifies what the anticipated scope of practice will be for the audiology technicians. With EHDI standing for Early Hearing Detection and Intervention, CHW for Community Healthcare Worker and CRW for Community Rehabilitation Worker, this guideline specifies the following in relation to audiology technicians (HPCSA, n.d., p. 4):

- Conduct promotion and prevention activities (e.g. hearing conservation programme, ear care)
- Screen (EHDI, ototoxicity, middle ear status) and identify clients at risk and with established risk for hearing and balance difficulties using basic screening audiology equipment
  ■ Otoscopic examination
  ■ Immittance measures
  ■ Pure tone (air conduction)
  ■ Otoacoustic emissions
  ■ Automated auditory brainstem response
  ■ Cerumen management
- Implement intervention plan and monitor progress of patients with hearing difficulties
- Facilitate support services (e.g. caregiver support groups)
- Follow-up care post hearing aid fittings
- Assist clients with (re-)integration (optimal participation and activity) into the community and return to work or school
- Liaise with:
  ■ All community structures and organizations including other primary health care service providers (e.g. CHW, CRW)
  ■ Other institutions

The author argues that, as the curriculum and registration of these cadres (audiology technicians) will be regulated by the HPCSA, and because they are to be supervised by audiologists during their practice, tele-audiology PSF activities can arguably be efficiently inserted into the audiology technician’s training and scope of practice. However, until this recommendation is in place, and perhaps, separately to this suggestion, careful planning by the South African audiology community is required around the competency and use of PSFs during tele-audiology to make sure that patients remain protected whilst best practice is applied. This is particularly important as current findings seem to indicate heterogeneity in the training.

Coco and Marrone (2020, p. 1) highlight four key considerations that audiologists planning on engaging in tele-audiology should keep in mind, and the current author suggests that these be the same areas under careful cognisance for PSFs: (1) ‘laws, regulations, and payment and coverage issues; (2) which services to provide; (3) hardware, software, and environmental considerations; and (4) patient safety and privacy’. These considerations should form part of the planning for PSFs, their training, as well as regulations surrounding their employment.

### Length of training and its implications

The earlier presented finding on significant preference for synchronous tele-audiology in the studies reviewed is consistent with the second finding from this scoping review, that of a short period of training documented for PSFs. Fundings indicate training that ranges from 1 h (Erkkola-Anttinen et al., 2019) to a maximum of 5 days (Eksteen et al., 2019; Ramkumar et al., 2018), again revealing the diversity in the character of the training practices. Because in synchronous tele-audiology, the audiologist is present via video connection, ongoing in-service training can occur and close monitoring of the PSF is possible. The current author, however, believes that the time frame for the training as reflected in the current review does not allow for extensive and comprehensive training, and limits both the breadth and depth of content covered, even for a paraprofessional, which the PSF can be argued to be. These recorded time frames might have been appropriate in the studies reviewed because the PSFs had very specific tasks assigned to them, and so restricted training based on the assigned tasks was therefore appropriate. This finding has implications for content and practical training for PSFs if asynchronous tele-audiology will also be included, as might be the case if audiology technicians are utilised and within hybrid models in the South African context.

### Diversity in the range of PSFs used and its implications for the training

The third finding from this scoping review reveals the diversity in the range of PSFs used, as they have a variety of backgrounds; it also has significant implications for the nature and type of training provided. In the current review, village and community health workers were the most common individuals who served as PSFs (Coco et al., 2021; Eksteen et al., 2019; Ramkumar et al., 2018; Ravi et al., 2020). This has been documented in South Africa, for example, in a study by Yousuf Hussein et al. (2018) on community-based hearing screening for young children using an mHealth service delivery model, where community health workers served as PSFs. In the current review, generally, healthcare facilitators (Lundberg et al., 2017), teachers (Monica et al., 2017), community members (Govender & Mars, 2018), audiologists or speech pathologists students and professionals (Hughes et al., 2018; Venail et al., 2018), audiometric technicians (Hatton et al., 2019), nurses (Coco et al., 2020) and parents (Erkkola-Anttinen et al., 2019) were used as PSFs, with the roles played and intensity of training appearing consistent with the documented PSFs. Bennett et al. (2020, p. 12) suggest use of patients themselves as PSFs and describe this as ‘self-management: where the client is actively involved in tasks that immediately or consequently affect their health’, with Thai-Van et al. (2021) putting this to clinical practice. The current author believes that the use of audiologists or speech pathologists as well as students in these professions as PSFs is not cost-effective nor sensible in the long run as it fails to achieve the goal of increasing access. Audiologists and audiology students should be the ones managing the tele-audiology programmes and supervising PSFs, particularly within the South African context where capacity versus demand challenges have been well documented (Khoza-Shangase & Sebothoma, 2022; Pillay et al., 2020). Therefore, the decision of who can serve as PSFs is an important one that should carefully consider contextual realities.

Generally, the scoping review results reveal that there is a heterogeneity, lack of regularisation, standardisation and structure in the training programmes adopted. This lack of uniformity in the training can negatively impact best practice and compromise patient care during tele-audiology applications. Tao et al. (2018) caution about the negative impact of inconsistent and non-standard or lacking training of PSFs. Only one of the reviewed studies detailed a training programme with structured content (Coco et al., 2021). Coco et al.’s (2021) study described their training as comprising three levels: (1) introductory level where topics on basic information about causes and effects of hearing loss were covered, as well as basic tele-audiology and its benefits, (2) intermediate level where the focus was on technology, roles of the tele-audiology service delivery team, as well as on tele-audiology technology and patient safety and confidentiality and (3) facilitator level where knowledge and hands-on skills required to serve as PSFs were the focus. This is also the only study from the review that reported that the PSFs training also included them attending a 4-h in-person observation at a university-based adult audiology clinic with details of the components of audiology observed at the clinic provided.

### Heterogeneity in the training

The content of the training documented in the reviewed studies, which was mainly provided by audiologists and physicians or otologists with appropriate scope-of-practice qualifications, can be grouped into five areas: (1) infection control (Monica et al., 2017), (2) patient and equipment management (e.g. placement of transducers, insertion of probes, positioning of otoscopes, orientation of equipment, handling, manipulating and using hearing aids and accessories, troubleshooting) (Govender & Mars, 2018; Lundberg et al., 2017; Monica et al., 2017; Ravi et al., 2020; Tao et al., 2021; Venail et al., 2018), (3) how to perform and evaluate certain tasks (e.g. conducting otoscopy, OAEs, ABR, conducting hearing screening, setting up the microphone and web camera for videoconferencing, connecting audiological equipment to the laptop) (Coco et al., 2021; Eksteen et al., 2019; Erkkola-Anttinen et al., 2019; Govender & Mars, 2018; Hatton et al., 2019; Hughes et al., 2018; Lundberg et al., 2017; Ramkumar et al., 2018; Ravi et al., 2020; Tao et al., 2021), (4) computer, technical and proper software use (e.g. clinical use smartphones, operating the laptop and ensuring proper connectivity of the computer hardware remotely, using the internet and using the mobile hotspot) (Coco et al., 2021; Monica et al., 2017; Ravi et al., 2020; Tao et al., 2021; Venail et al., 2018) and (5) working and connecting remotely, with very limited reports on training on anatomy of the ear such as Venail et al. (2018), who covered anatomy of the external and middle ear in their training. These areas, with the exclusion of those specific to remote connectivity, mirror those included in the audiology technician’s scope of practice, thus supporting the recommendation by the current author that this role be included in those paraprofessionals’ training (HPCSA, n.d.).

## Conclusion

Current findings revealing heterogeneity in the training provided to PSFs highlight the need for careful interrogation of this core aspect of tele-audiology during COVID-19 and beyond, where COVID-19 has thrust this model of service delivery to the forefront. When planned and implemented properly, this planning can have a significantly positive impact in the provision of ear and hearing care for the African context, with possibilities of contextually relevant and responsive service provision where PSFs are drawn from the same populations where the remote service is being provided, enhancing linguistic and cultural diversity considerations in clinical care (Khoza-Shangase & Mophosho, 2018, 2021). As valuable as PSFs are to the application of tele-audiology, access to them can be just as challenging as access to audiologists within the African context (Biagio, Swanepoel, Laurent, & Lundberg, 2014; Crowell, Givens, Jones, Brechtelsbauer, & Yao, 2011), hence the importance of careful planning around who can serve in this role as well as clarifying the nature and extent of training required. The limited published evidence on the training of PSFs highlights this gap in practice and research, hence the importance of current and future planning. This planning should also consider cost implications that are related to human resource regulations, including issues around medical aid coverage should this service be reimbursable. Lastly, standardised training of PSFs will ensure that these cadres possess the same minimum competency levels, allowing for easy, fair and transparent accreditation and registration processes for adherence to regulations and policies governing healthcare provision in South Africa. Quality standards within best practice models in ear and hearing care can be maintained and patient safety safeguarded.
